# Performance with a new bone conduction implant audio processor in patients with single-sided deafness

**DOI:** 10.1007/s00405-023-07852-x

**Published:** 2023-01-24

**Authors:** Wilhelm Wimmer, Michael Zbinden, Tom Gawliczek, Alexander Huber, Marco Caversaccio, Martin Kompis

**Affiliations:** 1 Department of ENT, Head and Neck Surgery, Bern University Hospital, University of Bern, Bern, Switzerland; 2 Hearing Research Laboratory, ARTORG Center, University of Bern, Bern, Switzerland; 3 Department of Otorhinolaryngology, Head and Neck Surgery, University Hospital Zurich, Zurich, Switzerland

**Keywords:** BONEBRIDGE, SAMBA 2 BB, Speech enhancement, Unilateral deafness, Speech understanding in noise

## Abstract

**Purpose:**

The SAMBA 2 BB audio processor for the BONEBRIDGE bone conduction implant features a new automatic listening environment detection to focus on target speech and to reduce interfering speech and background noises. The aim of this study was to evaluate the audiological benefit of the SAMBA 2 BB (AP2) and to compare it with its predecessor SAMBA BB (AP1).

**Methods:**

Prospective within-subject comparison study. We compared the aided sound field hearing thresholds, speech understanding in quiet (Freiburg monosyllables), and speech understanding in noise (Oldenburg sentence test) with the AP1 and AP2. Each audio processor was worn for 2 weeks before assessment and seven users with single-sided sensorineural deafness (SSD) participated in the study. For speech understanding in noise, two complex noise scenarios with multiple noise sources including single talker interfering speech were used. The first scenario included speech presented from the front (S_0_N_MIX_), while in the second scenario speech was presented from the side of the implanted ear (S_IPSI_N_MIX_). In addition, subjective evaluation using the SSQ12, APSQ, and the BBSS questionnaires was performed.

**Results:**

We found improved speech understanding in quiet with the AP2 compared to the AP1 aided condition (on average + 17%, *p* = 0.007). In both noise scenarios, the AP2 lead to improved speech reception thresholds by 1.2 dB (S_0_N_MIX_, *p* = 0.032) and 2.1 dB (S_IPSI_N_MIX_, *p* = 0.048) compared to the AP1. The questionnaires revealed no statistically significant differences, except an improved APSQ usability score with the AP2.

**Conclusion:**

Clinicians can expect that patients with SSD will benefit from the SAMBA 2 BB by improved speech understanding in both quiet and in complex noise scenarios, when compared to the older SAMBA BB.

**Supplementary Information:**

The online version contains supplementary material available at 10.1007/s00405-023-07852-x.

## Introduction

Patients with single-sided sensorineural deafness (SSD) experience communication difficulties resulting from reduced intelligibility for speech from the side of the deaf ear, and impaired speech understanding in noise [1]. Cochlear implants are one therapeutic option showing good clinical benefit [2]. However, in cases where cochlear implantation is not an option, CROS hearing aids or bone conduction hearing systems can be used to overcome the acoustic head shadow and to improve speech understanding in certain noisy situations [3, 4]. A limitation of CROS hearing aids and bone conduction systems in SSD patients is that restoration of binaural hearing is not possible [5, 6].

One option available is the BONEBRIDGE system (MED-EL, Innsbruck, Austria), an active transcutaneous bone conduction implant [7]. It is indicated for the treatment of patients with SSD as well as conductive or mixed hearing loss [8–12]. Its latest audio processor, the SAMBA 2 BB (AP2), offers more frequency and compression bands than its predecessor, the SAMBA BB (AP1). Additionally, the AP2 features a new automatic listening environment detection to focus on target speech and to reduce interfering speech and background noises in complex acoustic scenarios [13]. The AP2 is available for the BONEBRIDGE system and also for the VIBRANT SOUNDBRIDGE (MED-EL) active middle ear implant. For the audiological performance of the AP2 with the SOUNDBRIDGE, a study with 15 patients was recently published by Rahne et al. [14]. The authors showed that in their cohort both audio processors resulted in comparable sound field hearing thresholds, but that with the AP2, better speech understanding in quiet and noise could be obtained, along with lower listening effort, and higher subjective satisfaction compared with the AP1. Since the maximum output power and gain are similar for both audio processors, they concluded that the improvements are due to the new signal processing of the AP2 [14]. In light of the first promising results by Rahne et al. [14], we wanted to investigate if the new audio processor could also provide benefits for SSD patients treated with bone conduction implants. This is clinically relevant, as hearing with the normal contralateral ear and bone conduction system must be expected to be considerably different from that of a sensorineural hearing loss and an implantable hearing aid.

Therefore, the aim of this study was to evaluate the audiological performance and subjective satisfaction of the AP2 compared to the AP1 in SSD patients with a BONEBRIDGE implant. We hypothesized that the advanced speech enhancement algorithm would result in improved speech reception thresholds in complex noise scenarios.

## Material and methods

### Study design and participants

We performed a prospective study with a repeated-measures design to compare the audiological performance of the older audio processor (AP1) with its successor model (AP2) in SSD patients. The study protocol was approved by our local institutional review board (KEK-BE 2020-02625). We included 7 German-speaking patients who received a BONEBRIGDE (BCI 601) and had at least 2 years of listening experience with their implant. The mean pure tone averages (PTA) over 0.5, 1, 2, and 4 kHz of the bone conduction thresholds were 6.8 dB HL (standard deviation; SD of 8.9 dB HL) for the contralateral ear. For the ipsilateral ear, the bone conduction thresholds were all above audiometric assessment levels (i.e., 65, 70, 75, and 80 dB HL for 0.5,1,2, and 4 kHz, respectively). The mean age of the patients was 44 years (min. 31 year; max. 54 year; 2 women and 5 men). All patients gave written informed consent before study participation.

### Study sessions and audio processor fitting

The patients served as their own control. In an initial visit, either the AP1 or AP2 was fitted according to a counter-balanced order. Following a familiarization period of 2 weeks, the first study session took place. After the session, the other audio processor was fitted and worn by the patients for at least 2 weeks before they were invited to the second study session. Audio processor fitting consisted of pure tone audiometry, Vibrogram assessment (integrated in-situ measurement in the fitting software), and fitting using the built-in SSD rule. The first fit setting was used by applying air conduction, bone conduction and Vibrogram thresholds at 100% acclimatization. Fine-tuning was performed after a short familiarization time. For the fitting, we used the SYMFIT software (versions 7.0/8.0; MED-EL, Innsbruck, Austria). The fitting and study all sessions took place in an acoustic chamber.

### Sound field hearing thresholds and speech understanding in quiet

Sound field hearing thresholds and speech understanding in quiet were assessed under unaided and aided conditions with a loudspeaker placed in front of the patients at a distance of 1 m. The contralateral (i.e., normal-hearing) ear was plugged and muffled during the measurements. We measured the sound field hearing thresholds (in dB hearing level; HL) with warble tones at 0.25, 0.5, 1, 2, 4, and 6 kHz. The word recognition scores (WRS) in quiet (in %) were assessed using German Freiburg monosyllabic test lists at 65 dB sound pressure level (SPL).

### Speech understanding in noise

**Fig. 1:**
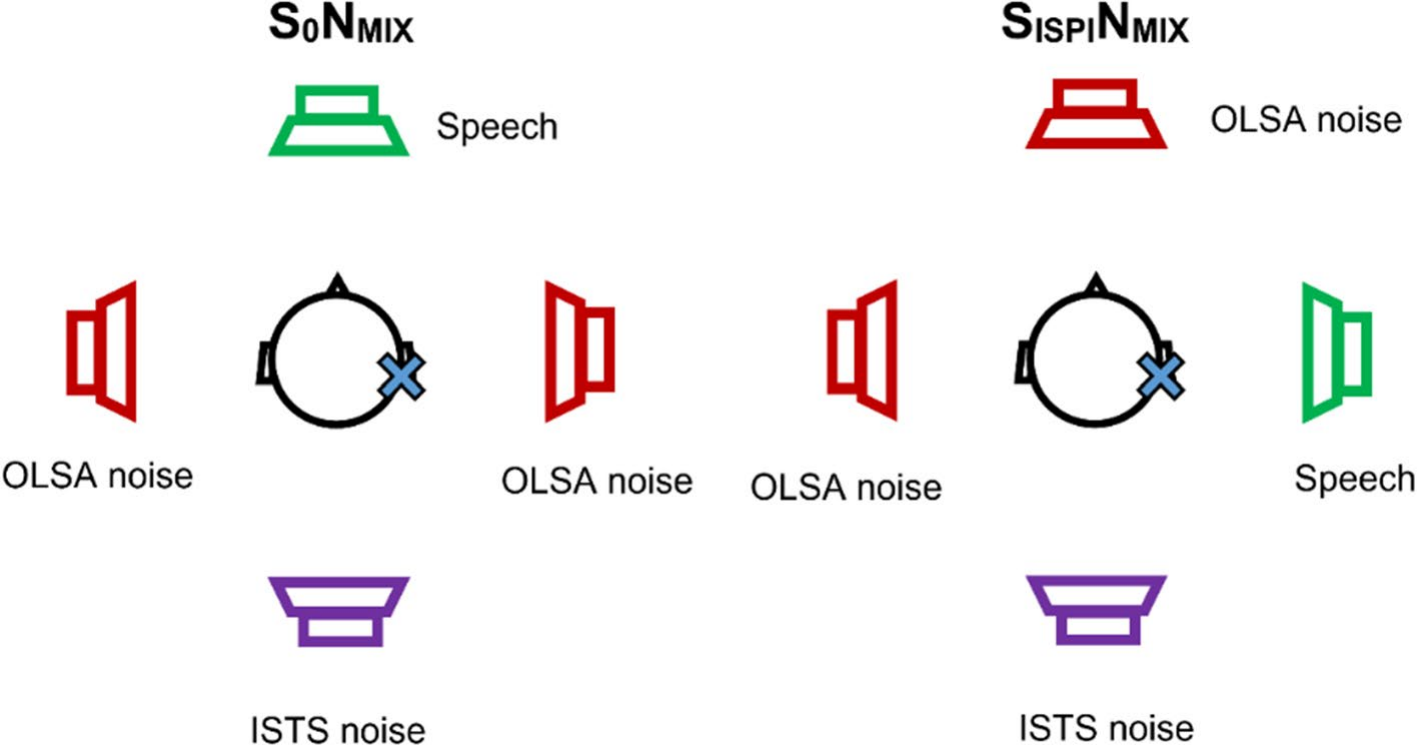
Test scenarios for speech understanding in noise. In both scenarios, mixed background noise was generated by continuously playing incoherent speech babble noise from 2 loudspeakers (OLSA noise), while an additional interfering speech-like noise (ISTS noise) was presented from the speaker behind the subjects. The test sentences were either played from the front (S_0_N_MIX_) or ipsilateral to the implant (S_IPSI_N_MIX_)

The speech reception threshold (SRT) in noise, i.e. the signal-to-noise ratio in dB necessary for 50% correct speech understanding (in dB SNR), was determined with an adaptive German matrix test (Oldenburg sentence test, OLSA). To simulate complex listening situations, two different test scenarios with 4 loudspeakers at 1 m distance to the patient were used (Fig. 1). Two incoherent speech babble (OLSA) noise sequences were continuously playing from either the sides or front. In addition, a non-stationary speech-like noise (International Speech Test Signal, ISTS) was presented from behind [15]. The total noise level was 65 dB SPL at the center point. In the first scenario, the test sentences were presented from the front (S_0_N_MIX_), while in the second scenario the sentences were played from the side ipsilateral to the implanted ear (S_IPSI_N_MIX_). The contralateral ear was open during testing. Two training lists were run and their results were discarded, to familiarize the patients with the test procedure. To minimize training and fatigue effects, the order of the aided conditions (i.e., UNAIDED, AP1, or AP2) as well as the test scenarios were systematically counter-balanced. The test sentence lists were randomly selected.

### Subjective evaluation

The patients evaluated their subjective benefit using the Speech, Spatial and Qualities of Hearing Scale (SSQ12), the Audio Processor Satisfaction Questionnaire (APSQ), and the Bern Benefit in Single-Sided Deafness (BBSS) questionnaire. The SSQ12 consists of 12 items rated on a visual analog scale ranging from 0 to 10 and is divided into 3 domains (speech, spatial, and qualities) [16]. The APSQ consists of 15 items of 3 domains (comfort, social life, and usability) and also ranges from 0 to 10 [17]. The Bern Benefit in Single-Sided Deafness (BBSS) comprises of 10 questions and the answers range from − 5 to + 5 [18].

### Statistical analysis

The audiological outcome was measured repeatedly under 3 aiding conditions (“Unaided”, “AP1”, and “AP2”). We used separate linear mixed effects models to test differences in sound field PTAs, WRS in quiet, and SRT in noise. The aiding condition was included as fixed effect, while the patient number served as random intercept. For post-hoc comparisons between aided conditions, Tukey all-pairs tests with Holm correction were performed. Questionnaire items were obtained for the aided conditions only (i.e., “AP1” and “AP2)”, and their differences were tested with the Wilcoxon singed rank test. We used R Studio (version 2022.07.1) with the "lme4", "multcomp", and “ggprism” packages installed to compute the statistics and generate the graphs.

## Results

### Sound field hearing thresholds

**Fig. 2:**
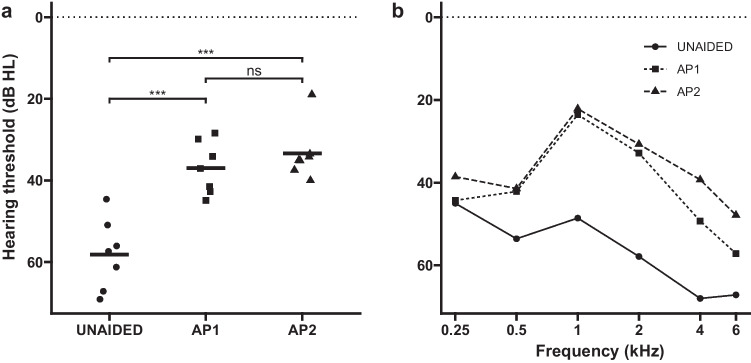
Sound field hearing thresholds for the test conditions expressed as **a** pure tone average (PTA) over 0.5, 1, 2, and 4 kHz and **b** averaged separately over different frequencies. *ns* non significant, ***p < 0.001

Figure 2 summarizes the individual sound filed thresholds (PTAs) for the different conditions. Both audio processors improved the sound field hearing thresholds, from an unaided PTA of 58.2 ± 8.5 dB HL to aided average levels of 37.0 ± 6.3 dB HL with the AP1 (*p* < 0.001) and 33.4 ± 6.8 dB HL with the AP2 (*p* < 0.001). No statistically significant PTA differences were observed between the audio processors (*p* = 0.30). A summary of the statistical model output is provided in the supplementary material.

### Speech understanding in quiet

**Fig. 3:**
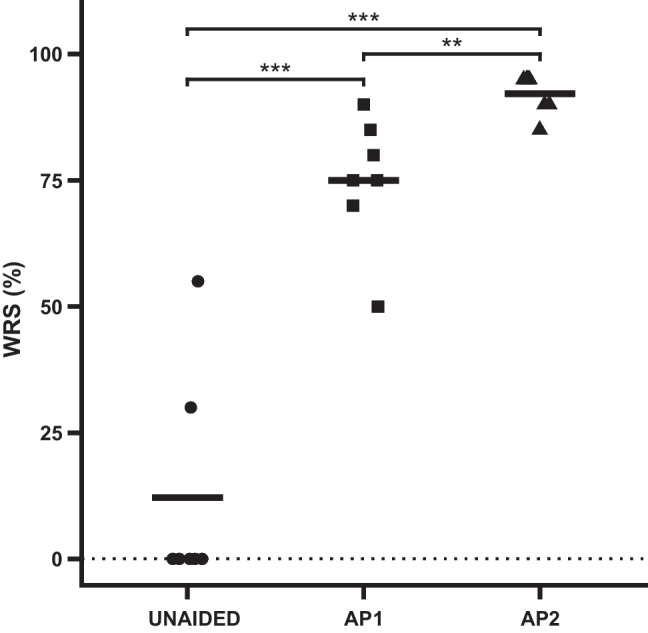
Word recognition score (WRS) in quiet for monosyllables at tested 65 dB SPL for all aiding conditions (contralateral ear plugged). **p < 0.01, ***p < 0.001

The results for speech understanding in quiet are shown in Fig. 3. The mean WRS in quiet was 12 ± 22% in the unaided condition and was improved with both devices, reaching 75 ± 13% with the AP1 (*p* < 0.001) and 92 ± 4% using the AP2 (*p* < 0.001). The 17% difference in scores between the AP1 and the AP2 is statistically significant (*p* = 0.007). A summary of the statistical model output is provided in the supplementary material.

### Speech understanding in noise

**Fig. 4:**
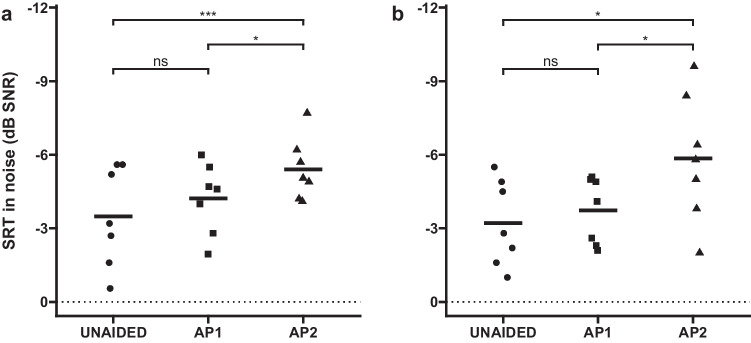
Speech reception thresholds (SRTs) in complex background noise with speech presented from **a** the front (S_0_N_MIX_, left figure) and **b** the side ipsilateral to the implanted ear (S_IPSI_N_MIX_). The contralateral ear was open during testing. *ns* non significant, *p < 0.05, ***p < 0.001

Speech reception thresholds in noise are shown in Fig. 4 for all conditions and test scenarios. In the S_0_N_MIX_ test scenario, an average SRT of − 3.5 ± 2.0 dB SNR was achieved in the unaided condition, while − 4.2 ± 3.9 dB SNR (*p* = 0.14) and − 5.4 ± 1.3 dB SNR (p < 0.001) were achieved with the AP1 and AP2, respectively. Post-hoc testing indicated that the difference of 1.2 dB between the AP1 and AP2 was statistically significant (*p* = 0.032).

In the S_IPSI_N_MIX_ test scenario, the patients achieved an average SRT of − 3.2 ± 1.8 dB SNR in the unaided condition, compared to − 3.7 ± 1.4 dB SNR (*p* = 0.59) and − 5.8 ± 2.6 dB SNR (*p* = 0.015) with the AP1 and AP2, respectively. In this test scenario, a tendency of improvement was observed between the AP2 and AP1 conditions (2.1 dB, *p* = 0.048). A summary of the statistical model output is provided in the supplementary material.

### Subjective evaluation

**Fig. 5:**
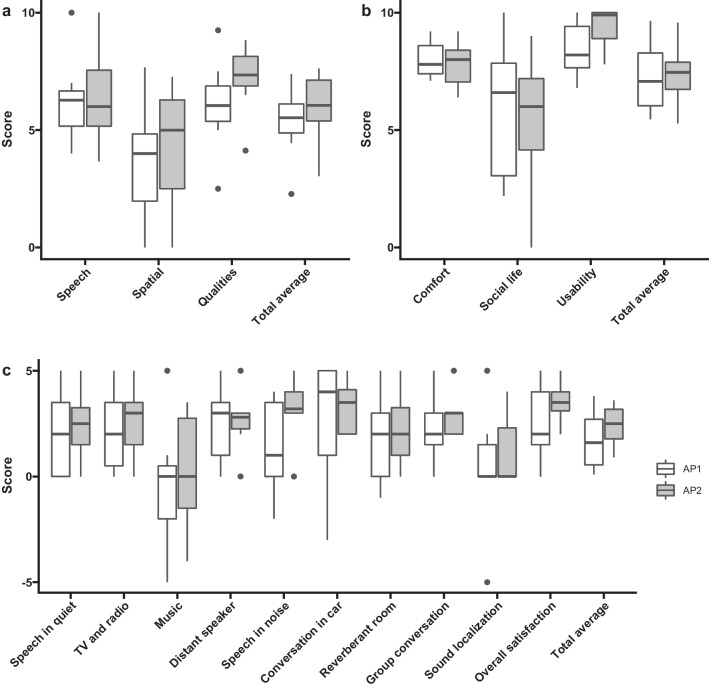
Subjective evaluation results for **a** the Speech, Spatial and Qualities of Hearing Scale (SSQ12), **b** the Audio Processor Satisfaction Questionnaire (APSQ), and **c** the Bern Benefit in Single-Sided Deafness (BBSS) questionnaire

The SSQ12 questionnaire showed a total average score of 5.3 ± 1.6 points, a speech subscale of 6.3 ± 2.0 points, a spatial subscale of 3.6 ± 2.7 points and a qualities subscale 6.3 ± 2.0 points using the AP1. With the AP2, the total average score was 6.0 ± 1.7 points, 6.4 ± 2.1 points in the speech subscale, 4.3 ± 2.7 points in the spatial subscale, and 7.2 ± 1.6 points in the qualities subscale. No statistically significant differences were observed (Fig. 5a). The AP1 scores of the APSQ were 8.0 ± 0.9 points, 5.8 ± 3.1 points, and 8.5 ± 1.2 points for the comfort, social life and usability subscales. After wearing the AP2, the participants rated the APSQ at 7.8 ± 1.0 points, 5.4 ± 3.0 points, and 9.4 ± 0.9 points for the comfort, social life and usability subscales. The usability subscale improved significantly with the AP2 (*p* = 0.036) compared to the AP1 (Fig. 5b).The BBSS questionnaire revealed no statistically significant differences between the test items (Fig. 5c). The overall score with the AP2 was higher, with the largest difference for the speech understanding in noise item.

## Discussion

Current advances in audio processor chip technology enable the implementation of new algorithms capable of handling increasingly complex listening situations. In each new processor generation, it is important that the effectiveness and audiological benefit of these algorithms is evaluated. As more advanced technologies become available, the test setups should be designed appropriately to avoid possible saturation effects and to ensure sensitivity and specificity to show treatment effects and differences. In this study, we compared the audiological benefit of the new AP2 with its predecessor. For well controlled, reproducible yet realistic settings for the speech in noise tests, we used complex listening scenarios including several incoherent noise sources and speech babble. Because the difference between the two processor compared were quite pronounced, we can draw a number of conclusions about the performance of the two processors despite the limited sample size.

### Speech understanding in quiet

Both audio processors showed substantial improvements in aided sound field hearing thresholds and speech understanding in quiet compared to the unaided condition. The AP2 had similar aided hearing thresholds as the AP1, although with the AP2, participants tended to have slightly better sound field threshold at higher frequencies (4 kHz and 6 kHz). Both devices have similar maximum power output and gain ranges, and the fitting of the audio processors were conducted according to the manufacturer’s recommendation. Somewhat surprisingly, the word recognition scores in quiet was on average 17% higher with the AP2 compared with the AP1. Our findings are very much in line with and support the findings of Rahne et al. [14], who reported similar aided hearing thresholds with both audio processors, higher output of the AP2 at frequencies above 4 kHz and, in addition, improved monosyllabic word recognition scores using the AP2. We agree with their suggestion that the improved signal processing capabilities of the AP2 are most probably the reason for the better audiological performance, although we cannot tell exactly which individual features cause these improvements.

### Speech understanding in noise

To test speech understanding in noise, we adapted the experimental setup used by Rahne et al. [14]. The first test scenarios correspond to the situation of a conversation in a crowed situation with a person in front of the user (S_0_N_MIX_). In this scenario, the ISTS from the back most probably confused the beamformer implementation of the AP1, resulting in a non-significant 0.7 dB improvement of the speech reception threshold only. In contrast, the algorithm implemented in the AP2 seems to be more capable to suppress disturbing voices from the background, reflected by a significant improvement of the speech reception threshold compared to the unaided and the aided AP1 conditions. Patients with SSD specifically face difficulties to understand speech from the side of the deafened ear. In this unfavorable situation, beamformers need to be able to focus on the side while suppressing noise from the front. This situation was tested in the S_IPSI_N_MIX_ scenario. Again, ISTS noise was presented from behind, rendering a challenging noise scenario for the AP1 audio processor and resulting in no significant improvement compared to the unaided condition. With the AP2, we observed improved speech reception thresholds compared to the unaided and the AP1 conditions. Thus, in these noise scenarios, users of AP2 seem to benefit from the new signal processing features.

### Subjective benefit

The subjective evaluation did not reveal any statistically significant differences between the AP1 and AP2, with the exception of a better usability rating in the APSQ. We assume that this could be due to the easier opening of the battery case. There was also the tendency of better scores in the SSQ12 qualities domain and the BBSS speech in noise and overall satisfaction items, however, not statistically significant.

### Study limitations

The biggest limitation of our study is the limited sample size. We were unable to find and to recruit more volunteers which met all inclusion criteria. Unfortunately, it is quite probable that the situation is not much different at other centers. Despite the limited sample size, we were able to find several statistically and clinically significant differences between the two processors. We were also able to confirm some findings from an earlier study [14]. However, it is possible that we have missed smaller effects. Most notably, we found almost no differences in the subjective evaluation. Common problems of CROS treatment solutions are excess noise and masking via the aid/implant in unfavorable situations [19]. The available technology in bone conduction and middle ear implantable devices are similar to those implemented in hearing aid technology [20]. In the context of the tested SSD cohort, we believe that the presented results are of relevance for the clinical work.

## Conclusion

Our SSD patient cohort fitted with the SAMBA 2 BB audio processor showed clinically relevant improvements in speech understanding in quiet and noise. We therefore conclude that SSD patients with a BONEBRIDGE implant can most probably benefit from an upgrade from the SAMBA BB to the SAMBA 2 BB audio processor.

## Supplementary Information

Below is the link to the electronic supplementary material.

Supplementary file1 (PDF 129 KB)

## Data Availability

Availability of data and material is made available on request.

## References

[1] Kompis M, Wimmer W, Caversaccio M (2017). Long term benefit of bone anchored hearing systems in single sided deafness. Acta Otolaryngol.

[2] Peter N, Kleinjung T, Probst R (2019). Cochlear implants in single-sided deafness–clinical results of a Swiss multicentre study. Swiss Med Wkly.

[3] Peters JP, Smit AL, Stegeman I (2015). Bone conduction devices and contralateral routing of sound systems in single-sided deafness. Laryngoscope.

[4] Wimmer W, Kompis M, Stieger C (2017). Directional microphone contralateral routing of signals in cochlear implant users: a within-subjects comparison. Ear Hear.

[5] Yang J, Wang Z, Huang M (2018). BoneBridge implantation in patients with single-sided deafness resulting from vestibular schwannoma resection: objective and subjective benefit evaluations. Acta Otolaryngol.

[6] Ghoncheh M, Lilli G, Lenarz T, Maier H (2016). Outer ear canal sound pressure and bone vibration measurement in SSD and CHL patients using a transcutaneous bone conduction instrument. Hear Res.

[7] Huber AM, Strauchmann B, Caversaccio M (2022). Multicenter results with an active transcutaneous bone conduction implant in patients with single-sided deafness. Otol Neurotol.

[8] Cywka KB, Skarzynski PH, Krol B, Hatzopoulos S, Skarzynski H (2022). Evaluation of the Bonebridge BCI 602 active bone conductive implant in adults: efficacy and stability of audiological, surgical, and functional outcomes. Eur Arch Otolaryngol.

[9] Ratuszniak A, Skarzynski PH, Gos E, Skarzynski H (2022). Self-Rated benefits of auditory performance after Bonebridge implantation in patients with conductive or mixed hearing loss, or single-sided deafness. Life.

[10] Cywka KB, Skarżyński H, Król B, Skarżyński PH (2021). The Bonebridge BCI 602 active transcutaneous bone conduction implant in children: objective and subjective benefits. J Clin Med.

[11] Wimmer W, Gerber N, Guignard J, Dubach P, Kompis M, Weber S, Caversaccio M (2015). Topographic bone thickness maps for Bonebridge implantations. Eur Arch Otorhinolaryngol.

[12] Król B, Cywka KB, Skarżyńska MB, Skarżyński PH (2021). Implantation of the Bonebridge BCI 602 after mastoid obliteration with S53P4 bioactive glass: a safe method of treating difficult anatomical conditions-preliminary results. Life.

[13] Fischer T, Caversaccio M, Wimmer W (2021). Speech signal enhancement in cocktail party scenarios by deep learning based virtual sensing of head-mounted microphones. Hear Res.

[14] Rahne T, Fröhlich L, Wagner L (2021). Speech perception and hearing effort using a new active middle ear implant audio processor. Eur Arch Otorhinolaryngol.

[15] Holube I, Fredelake S, Vlaming M, Kollmeier B (2010). Development and analysis of an international speech test signal (ISTS). Int J Aud.

[16] Noble W, Jensen NS, Naylor G (2013). A short form of the speech, spatial and qualities of hearing scale suitable for clinical use: The SSQ12. Int J Aud.

[17] Billinger-Finke M, Bräcker T, Weber A (2020). Development and validation of the audio processor satisfaction questionnaire (APSQ) for hearing implant users. Int J Aud.

[18] Kompis M, Pfiffner F, Krebs M, Caversaccio M (2011). Factors influencing the decision for Baha in unilateral deafness: the Bern benefit in single-sided deafness questionnaire. In Implantable bone conduction hearing aids (Vol. 71, pp. 103–111). 10.1159/000323591 10.1159/000323591 21389710

[19] Arndt S, Laszig R, Aschendorff A (2017). Cochlear implant treatment of patients with single-sided deafness or asymmetric hearing loss. HNO.

[20] Postert B, Bicego L, Lochner J, Mojallal H (2022) The SAMBA 2 Audio Processor – Technical Facts and Initial Audiological Results. https://www.medel.pro/online-resources/white-papers. Accessed 13 Jan 2023.

