# Supplementary material for: Performance with a new bone conduction implant audio processor in patients with single-sided deafness
Source: Eur Arch Otorhinolaryngol. 2023 Jan 24;280(8):3585–91. doi: 10.1007/s00405-023-07852-x (PMC10313841; doi:10.1007/s00405-023-07852-x)
Supplement: Supplementary file 1 — Supplementary file1 (PDF 129 KB) [file 405_2023_7852_MOESM1_ESM.pdf]

SAMBA 2 Statistics

PTA

```
Linear mixed model fit by REML. t-tests use Satterthwaite's method
['lmerModLmerTest']
Formula: PTA4 ~ AP + (1 | ID)
   Data: SAMBA_DATA

REML criterion at convergence: 127.6

Scaled residuals:
     Min       1Q   Median       3Q      Max
-1.91000 -0.48728  0.00858  0.61919  1.29092

Random effects:
 Groups   Name        Variance Std.Dev.
 ID       (Intercept) 11.63    3.411
 Residual             41.39    6.434
Number of obs: 21, groups:  ID, 7

Fixed effects:
            Estimate Std. Error      df t value Pr(>|t|)
(Intercept)   58.214      2.752  16.420  21.151 2.41e-13 ***
APSAMBA      -21.250      3.439  12.000  -6.179 4.73e-05 ***
APSAMBA2     -24.821      3.439  12.000  -7.218 1.06e-05 ***
---
Signif. codes:  0 '***' 0.001 '**' 0.01 '*' 0.05 '.' 0.1 ' ' 1

Correlation of Fixed Effects:
         (Intr) APSAMBA
APSAMBA  -0.625
APSAMBA2 -0.625  0.500


Simultaneous Tests for General Linear Hypotheses

Multiple Comparisons of Means: Tukey Contrasts


Fit: lmer(formula = PTA4 ~ AP + (1 | ID), data = SAMBA_DATA)

Linear Hypotheses:
                      Estimate Std. Error z value Pr(>|z|)
SAMBA - UNAIDED == 0   -21.250      3.439  -6.179 1.29e-09 ***
SAMBA2 - UNAIDED == 0  -24.821      3.439  -7.218 1.59e-12 ***
SAMBA2 - SAMBA == 0     -3.571      3.439  -1.039    0.299
---
Signif. codes:  0 '***' 0.001 '**' 0.01 '*' 0.05 '.' 0.1 ' ' 1
(Adjusted p values reported -- holm method)
```

SPEECH IN QUIET

```
Linear mixed model fit by REML. t-tests use Satterthwaite's method
['lmerModLmerTest']
Formula: MONOSYL ~ AP + (1 | ID)
   Data: SAMBA_DATA

REML criterion at convergence: 151.9

Scaled residuals:
     Min       1Q   Median       3Q      Max
-1.31969 -0.77054  0.07498  0.25616  2.53495

Random effects:
 Groups   Name        Variance Std.Dev.
 ID       (Intercept)  81.55    9.03
 Residual             139.88   11.83
Number of obs: 21, groups:  ID, 7

Fixed effects:
            Estimate Std. Error     df t value Pr(>|t|)
(Intercept)   12.143      5.624 14.159   2.159   0.0485 *
APSAMBA       62.857      6.322 12.000   9.943 3.81e-07 ***
APSAMBA2      80.000      6.322 12.000  12.654 2.67e-08 ***
---
Signif. codes:  0 '***' 0.001 '**' 0.01 '*' 0.05 '.' 0.1 ' ' 1

Correlation of Fixed Effects:
         (Intr) APSAMBA
APSAMBA  -0.562
APSAMBA2 -0.562  0.500


        Simultaneous Tests for General Linear Hypotheses

Multiple Comparisons of Means: Tukey Contrasts


Fit: lmer(formula = MONOSYL ~ AP + (1 | ID), data = SAMBA_DATA)

Linear Hypotheses:
                      Estimate Std. Error z value Pr(>|z|)
SAMBA - UNAIDED == 0    62.857      6.322   9.943  < 2e-16 ***
SAMBA2 - UNAIDED == 0   80.000      6.322  12.654  < 2e-16 ***
SAMBA2 - SAMBA == 0     17.143      6.322   2.712  0.00669 **
---
Signif. codes:  0 '***' 0.001 '**' 0.01 '*' 0.05 '.' 0.1 ' ' 1
(Adjusted p values reported -- holm method)
```

SPEECH IN NOISE S0NMIX

```
Linear mixed model fit by REML. t-tests use Satterthwaite's method
['lmerModLmerTest']
Formula: SRTS0 ~ AP + (1 | ID)
   Data: SAMBA_DATA

REML criterion at convergence: 65.8

Scaled residuals:
     Min       1Q   Median       3Q      Max 
-1.42189 -0.57551 -0.05422  0.47772  1.45963 

Random effects:
 Groups   Name        Variance Std.Dev.
 ID       (Intercept) 1.7438   1.3205  
 Residual             0.8492   0.9215  
Number of obs: 21, groups:  ID, 7

Fixed effects:
            Estimate Std. Error      df t value Pr(>|t|)    
(Intercept)  -3.4929     0.6086  9.4513  -5.739 0.000233 ***
APSAMBA      -0.7286     0.4926 12.0000  -1.479 0.164877    
APSAMBA2     -1.9143     0.4926 12.0000  -3.886 0.002163 ** 
---
Signif. codes:  0 '***' 0.001 '**' 0.01 '*' 0.05 '.' 0.1 ' ' 1

Correlation of Fixed Effects:
         (Intr) APSAMBA
APSAMBA  -0.405        
APSAMBA2 -0.405  0.500 


Simultaneous Tests for General Linear Hypotheses

Multiple Comparisons of Means: Tukey Contrasts

Fit: lmer(formula = SRTS0 ~ AP + (1 | ID), data = SAMBA_DATA)

Linear Hypotheses:
                      Estimate Std. Error z value Pr(>|z|)    
SAMBA - UNAIDED == 0   -0.7286     0.4926  -1.479 0.139112    
SAMBA2 - UNAIDED == 0  -1.9143     0.4926  -3.886 0.000305 ***
SAMBA2 - SAMBA == 0    -1.1857     0.4926  -2.407 0.032153 *  
---
Signif. codes:  0 '***' 0.001 '**' 0.01 '*' 0.05 '.' 0.1 ' ' 1
(Adjusted p values reported -- holm method)
```

SPEECH IN NOISE SIPSINMIX

```
Linear mixed model fit by REML. t-tests use Satterthwaite's method
['lmerModLmerTest']
Formula: SRTSIPSI ~ AP + (1 | ID)
   Data: SAMBA_DATA

REML criterion at convergence: 80.7

Scaled residuals: 
     Min       1Q   Median       3Q      Max 
-1.85477 -0.53367  0.02611  0.44385  1.74848 

Random effects:
 Groups   Name        Variance Std.Dev.
 ID       (Intercept) 0.7839   0.8854  
 Residual             3.1061   1.7624  
Number of obs: 21, groups:  ID, 7

Fixed effects:
            Estimate Std. Error      df t value Pr(>|t|)    
(Intercept)  -3.2143     0.7455 16.6479  -4.312 0.000494 ***
APSAMBA      -0.5143     0.9421 12.0000  -0.546 0.595124    
APSAMBA2     -2.6429     0.9421 12.0000  -2.805 0.015884 *  
---
Signif. codes:  0 '***' 0.001 '**' 0.01 '*' 0.05 '.' 0.1 ' ' 1

Correlation of Fixed Effects:
         (Intr) APSAMBA
APSAMBA  -0.632        
APSAMBA2 -0.632  0.500 

Simultaneous Tests for General Linear Hypotheses


Multiple Comparisons of Means: Tukey Contrasts


Fit: lmer(formula = SRTSIPSI ~ AP + (1 | ID), data = SAMBA_DATA)

Linear Hypotheses:
                      Estimate Std. Error z value Pr(>|z|)  
SAMBA - UNAIDED == 0   -0.5143     0.9421  -0.546   0.5851  
SAMBA2 - UNAIDED == 0  -2.6429     0.9421  -2.805   0.0151 *
SAMBA2 - SAMBA == 0    -2.1286     0.9421  -2.260   0.0477 *
---
Signif. codes:  0 '***' 0.001 '**' 0.01 '*' 0.05 '.' 0.1 ' ' 1
(Adjusted p values reported -- holm method)
```
